# Spindle Cell Carcinoma of the Larynx: A Confusing Diagnosis for the Pathologist and Clinician

**DOI:** 10.1155/2015/831835

**Published:** 2015-12-15

**Authors:** Asli Bostanci, Gulay Ozbilim, Murat Turhan

**Affiliations:** ^1^Department of Otolaryngology-Head and Neck Surgery, Akdeniz University School of Medicine, 07059 Antalya, Turkey; ^2^Department of Pathology, Akdeniz University School of Medicine, 07059 Antalya, Turkey

## Abstract

Laryngeal spindle cell carcinoma (SpCC) is an uncommon subtype of squamous cell carcinoma which represents 0.5% of all laryngeal squamous cell carcinomas. It is a biphasic tumor consisting of the combination of a malignant mesenchymal spindle cell component and a squamous cell component that includes dysplasia, carcinoma in situ, or invasive carcinoma. Although it has aggressive biological features, the probability of making a diagnosis in the early stages is high as it often leads to obstructive symptoms in the early period. Due to its low incidence, there is no clear consensus on prognostic factors and optimal treatment strategies yet. In this paper, a 60-year-old laryngeal SpCC case that was effectively treated with wide local excision followed by adjuvant radiotherapy was presented with the literature.

## 1. Introduction

Spindle cell carcinoma (SpCC) is an uncommon subtype of squamous cell carcinoma. Morphologically, it is a biphasic tumor consisting of a squamous cell carcinoma in situ and/or invasively and a malignant spindle cell component with a mesenchymal phenotype [1]. Due to the heterogeneous and complex nature of the tumor, it was reported under various terms such as carcinosarcoma, sarcomatoid carcinoma, collision tumor, pleomorphic carcinoma, and pseudosarcoma. The improvements in our understanding of monoclonal epithelial origin of these tumors finally led to the proposal of using the term “spindle cell carcinoma” [2].

SpCC is observed mainly in the larynx, especially in the glottic region. Its incidence is 0.023 per 100,000, and it represents 0.5% of all laryngeal squamous cell carcinomas [3]. The tumor occurs usually as a smooth-surfaced polypoid mass that arises from the true vocal cord and anterior commissure [4]. Although it has an aggressive biological character, the probability of making a diagnosis in the early stages is high as it often leads to obstructive symptoms such as hoarseness, snoring, and coughing in the early period [5]. Due to its low incidence, there is no clear consensus on prognostic factors and optimal treatment strategies yet.

In this paper, a laryngeal SpCC case that was effectively treated with wide local excision followed by adjuvant radiotherapy was presented with the literature.

## 2. Case Presentation

The present study was conducted in accordance with the Declaration of Helsinki and was approved by the Ethics Committee of the Akdeniz University School of Medicine (Antalya, Turkey). Written informed consent was obtained from the patient.

**Figure 1:**
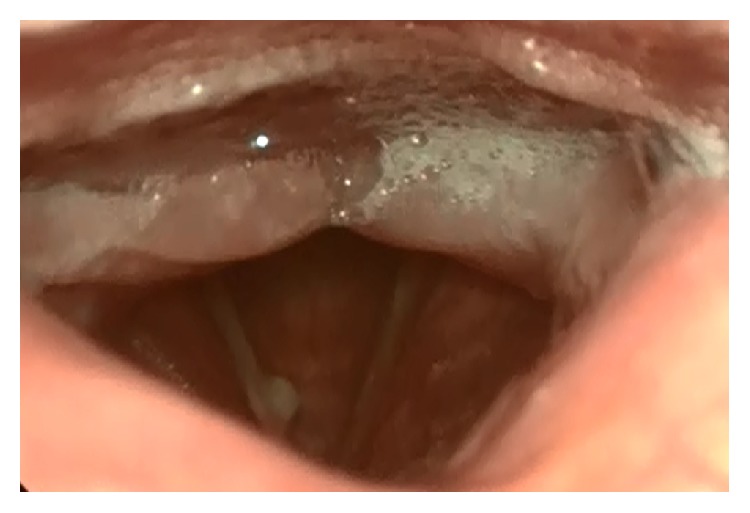
Endoscopic view of the submucosal nodular lesion with a smooth surface that is located in the middle of the right vocal cord.

A 60-year-old male patient was referred to the Akdeniz University School of Medicine with the complaint of chronic hoarseness that has existed for five years and noticeably increased in the last year. He had a history of 40 pack-years of cigarette smoking. Multiple medications prescribed by several different specialists due to diagnosis of vocal cord nodule failed to improve the symptoms. Although the head and neck examination was unremarkable, endoscopic examination revealed a submucosal nodular lesion with a smooth surface that was located in the middle of the right vocal cord (Figure 1).

**Figure 2:**
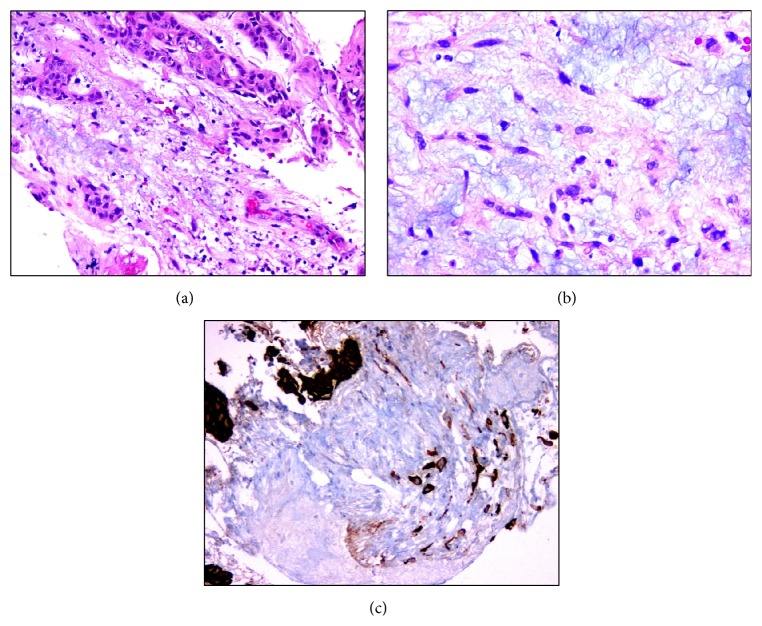
Microscopic appearance of atypical spindle cells and squamous cell carcinoma areas. (a) Hematoxylin and eosin (H&E), (magnification, ×20), (b) H&E (magnification, ×40), and (c) cytokeratin (magnification, ×20).

The laryngeal nodule was totally excised for pathological examination under direct laryngoscopy. Microscopic examination of the tumor revealed atypical spindle cells that were dispersed in a desmoplastic stroma under an ulcerated surface epithelium with fibrinoid necrosis and invasive tumor foci of squamous cell carcinoma (Figure 2). Epithelial component of the tumor exhibited strong immunoreactivity for cytokeratin and p63. Surgical margins were negative for tumor infiltration, but focally close, with tumor less than 2 mm from the posterior margin.

Adjuvant radiotherapy was administered due to close surgical margins while metastatic work-up with positron emission tomography combined with computed tomography was negative for systemic dissemination. The patient is now in the 12th month of his follow-up after a complete clinical response to primary therapy.

## 3. Discussion

SpCC is an aggressive malignant tumor typically observed in upper aerodigestive tract, in which nearly half of the cases are of larynx origin [6]. It was first reported in the literature in 1933 by Figi, under the name of “larynx sarcoma” [7]. According to recent population-based analysis data, a total of 312 cases of laryngeal SpCC were diagnosed in the United States of America between 1973 and 2011 [3].

Macroscopically, the tumor mostly has a polypoid appearance but may rarely occur as an infiltrative ulcerative lesion [1]. It is diagnosed by histologically showing the combination of a malignant mesenchymal spindle cell component that can contain homologous or heterologous elements and a surface squamous cell component that includes dysplasia, carcinoma in situ, or invasive carcinoma [1]. Epithelial and mesenchymal components can be present either separately or in a nested form while the spindle cell component usually constitutes the larger part of the tumor [8].

The heterogeneous morphological structure of SpCC poses significant diagnostic difficulties. The pathological differential diagnosis includes squamous cell carcinoma, leiomyosarcoma, fibrosarcoma, rhabdomyosarcoma, malignant melanoma, and inflammatory myofibroblastic tumor [9]. The biopsy samples that are taken superficially or in small pieces can lead to missing one of the components of the biphasic tumor and therefore misdiagnosis of SpCC as squamous cell carcinoma or sarcoma. In some cases, the differential diagnosis may not be possible even with the totally resected specimens without immunohistochemistry.

The cytokeratin, which has been reported to be expressed in 40–85% of the cases, is the most sensitive immunohistochemical marker that shows the epithelial component of the SpCC [5]. Epithelial membrane antigen (EMA) and carcinoembryonic antigen are other epithelial markers that exhibit variable immunoreactivity in these tumors [9]. Mesenchymal component of the SpCC frequently exhibits positive immunostaining with vimentin, which is also an epithelial marker [1]. Such a staining pattern supports the idea that these tumors are of epithelial origin and the sarcomatous spindle cells occur as a result of mesenchymal metaplasia [2]. Immunostaining with vimentin may also be positive for leiomyosarcomas. However, leiomyosarcoma cells show negative immunoreactivity for cytokeratins and positive immunoreactivity for muscle-specific actin [9].

Immunohistochemical studies usually allow reliable discrimination of SpCCs and sarcomas. On the other hand, it may lead to ambiguous results in some cases where the tumor lacks specific immunoreactivity. In such cases, the p63, which is a transcription factor of epidermal stem cell origin, can be beneficial in the discrimination of SpCCs from sarcomas. In a study assessing the diagnostic utility of several immunohistochemical markers of epithelial differentiation including p63, MOC-31, and thyroid transcription factor-1 on SpCCs, Lewis Jr. et al. [10] reported that staining for p63 had the greatest diagnostic utility. While immunostaining with p63 was positive in 63% of SpCCs, it was negative in the control group of 73 various primary and metastatic sarcomas, melanomas, and benign spindle cell lesions. The p63 stains were positive in many cases where immunohistochemistry was negative for both pan-cytokeratin and EMA.

Electron microscopy is another diagnostic tool used in the differential diagnosis of SpCCs with sarcomas [11, 12]. Although its use has declined significantly with the use of immunohistochemical stains, it may still have a role in tumors with inconclusive staining pattern. While the SpCCs have the combined epithelial and myofibroblastic features of desmosomes, tonofibrils, and filaments with dense bodies, fibrosarcomas consist largely of fibroblastic cells and leiomyosarcomas are packed in forming small cell groups with constant junctional complexes of nexus and zonula adherens types [12].

Ruling out a laryngeal mesenchymal malignancy is of critical importance in the differential diagnosis of SpCCs, since these tumors have a different histogenesis, natural history, biological behavior, and prognosis. Dubal et al. [3] reported that the majority of laryngeal SpCC cases have T1 (62%) and stage I (61%) tumors at presentation. Lymph node metastasis may occur (12.6%) but distant metastasis is uncommon (3.7%). The surgery yields a favorable prognosis when compared to radiotherapy. The 5-year disease-specific survival (DSS) is 84.0% with surgery alone, 84.2% with surgery combined with radiotherapy, and 60.5% with radiotherapy alone (*P* < 0.012). In addition, a glottic location of the tumor, as opposed to a nonglottic location, is significantly associated with improved prognosis (5-year DSS, 84.0% versus 51.9%, *P* < 0.0001). Compared to other laryngeal malignancies, SpCCs have intermediate (5-year) survival benefit (74.1% versus 64.6%, *P* < 0.020), but short-term (1-year, 90.9% versus 88%) and long-term (10-year, 57.9% versus 50.6%) survival rates are similar. Likewise, Thompson et al. [5] reported that a wide local excision should be the treatment of choice in most SpCC patients as these tumors are mostly of polypoid or pedicular nature and do not invade the stroma especially in the early period. The authors also suggested that radiotherapy might be used in the existence of recurrence or stromal invasion. On the other hand, in a retrospective study assessing the outcomes of early-stage (T1-T2) glottic SpCC cases (*n* = 28) treated with primary radiotherapy, Ballo et al. [13] reported that these patients had similar local-control rates to irradiated patients with similar volume disease with the more typical squamous cell carcinoma. Therefore, the authors suggested that the histologic diagnosis of SpCC instead of squamous cell carcinoma should not alter treatment recommendations in patients with early-stage disease.

In conclusion, laryngeal SpCC is an unusual occurrence that can be mostly diagnosed in the early stages despite its aggressive histological features. A wide local excision alone is mostly sufficient for definitive treatment in the early-stage disease. Adjuvant radiotherapy may be beneficial in local control of the disease in patients with close surgical margins.

## References

[1] Cardesa A., Zidar N., Barnes L., Eveson J. W., Reichart P., Sidransky D. (2005). Spindle cell carcinoma. *World Health Organization Classification of Tumours, Pathology & Genetics, Head and Neck Tumours*.

[2] Torenbeek R., Hermsen M. A. J. A., Meijer G. A., Baak J. P. A., Meijer C. J. L. M. (1999). Analysis by comparative genomic hybridization of epithelial and spindle cell components in sarcomatoid carcinoma and carcinosarcoma: histogenetic aspects. *The Journal of Pathology*.

[3] Dubal P. M., Marchiano E., Kam D. (2015). Laryngeal spindle cell carcinoma: a population-based analysis of incidence and survival. *The Laryngoscope*.

[4] Barnes L., Barnes L. (2009). Diseases of the larynx, hypopharynx, and trachea. *Surgical Pathology of the Head and Neck*.

[5] Thompson L. D. R., Wieneke J. A., Miettinen M., Heffner D. K. (2002). Spindle cell (sarcomatoid) carcinomas of the larynx: a clinicopathologic study of 187 cases. *The American Journal of Surgical Pathology*.

[6] Gerry D., Fritsch V. A., Lentsch E. J. (2014). Spindle cell carcinoma of the upper aerodigestive tract: an analysis of 341 cases with comparison to conventional squamous cell carcinoma. *Annals of Otology, Rhinology and Laryngology*.

[7] Figi F. A. (1933). Sarcoma of the larynx. *Archives of Otolaryngology: Head and Neck Surgery*.

[8] Lewis J. E., Olsen K. D., Sebo T. J. (1997). Spindle cell carcinoma of the larynx: review of 26 cases including DNA content and immunohistochemistry. *Human Pathology*.

[9] Marioni G., Bottin R., Staffieri A., Altavilla G. (2003). Spindle-cell tumours of the larynx: diagnostic pitfalls. A case report and review of the literature. *Acta Oto-Laryngologica*.

[10] Lewis J. S., Ritter J. H., El-Mofty S. (2005). Alternative epithelial markers in sarcomatoid carcinomas of the head and neck, lung, and bladder-p63, MOC-31, and TTF-1. *Modern Pathology*.

[11] Balercia G., Bhan A. K., Dickersin G. R. (1995). Sarcomatoid carcinoma: an ultrastructural study with light microscopic and immunohistochemical correlation of 10 cases from various anatomic sites. *Ultrastructural Pathology*.

[12] Nakanishi I., Katsuda S., Ooi A., Kajikawa K., Matsubara F. (1983). Diagnostic aspect of spindle-cell sarcomas by electron microscopy. *Acta Pathologica Japonica*.

[13] Ballo M. T., Garden A. S., El-Naggar A. K. (1998). Radiation therapy for early stage (T1-T2) sarcomatoid carcinoma of true vocal cords: outcomes and patterns of failure. *Laryngoscope*.

